# Effect of focusing flow on stationary spot machining properties in elastic emission machining

**DOI:** 10.1186/1556-276X-8-237

**Published:** 2013-05-16

**Authors:** Yoshinori Takei, Hidekazu Mimura

**Affiliations:** 1 Department of Precision Engineering, The University of Tokyo, 7-3-1 Hongo, Bunkyo-ku, Tokyo 113-8656, Japan

**Keywords:** Elastic emission machining, Fluid simulation, Focusing flow, Figure correction, Stationary spot machining

## Abstract

Ultraprecise optical elements are applied in advanced optical apparatus. Elastic emission machining (EEM) is one of the ultraprecision machining methods used to fabricate shapes with 0.1-nm accuracy. In this study, we proposed and experimentally tested the control of the shape of a stationary spot profile by introducing a focusing-flow state between the nozzle outlet and the workpiece surface in EEM. The simulation results indicate that the focusing-flow nozzle sharpens the distribution of the velocity on the workpiece surface. The results of machining experiments verified those of the simulation. The obtained stationary spot conditions will be useful for surface processing with a high spatial resolution.

## Background

Optical devices operating in extremely short wavelength ranges require unprecedented accuracy because a small figure error and/or slight surface roughness distorts the wavefront of the reflected light. In the field of precision machining, the degree of accuracy has been increased to atomic order. Various types of mirror or lens having a peak-to-valley (p-v) accuracy of 1 nm can now be fabricated, which are applied to the advanced optical apparatus used in X-ray microscopy and extreme ultraviolet lithography (EUVL) [1]. Ion beam figuring [2], magnetorheological finishing [3], and elastic emission machining (EEM) [4] are employed to process surfaces with atomic-order controllability. A surface profiler also plays a crucial role because figure correction is performed on the basis of measured data when the target accuracy is higher than 100 nm (p-v) [4].

In processing using profile data, the dwelling time of the spot profile is a parameter used to control the removal depth. The dwelling time distributions are converted to the scanning speed distributions of machining stages. The characteristics of the stationary spot such as the size, removal rate, and repeatability basically determine the performance of figure correction. The size of the spot and the removal rate are directly related to the spatial resolution and machining time, respectively, in figure correction. The high repeatability of the characteristics reduces the number of cycles between machining and measurements until the required accuracy is achieved.

EEM is one of the ultraprecision machining methods used to fabricate shapes with 0.1-nm accuracy without causing any crystallographic damage. A numerically controlled machining system has been developed for EEM [4]. The relationship between the surface morphology of particles and the microroughness of EEM surfaces was investigated using perfectly spherical particles [5]. The EEM surfaces were observed by scanning tunneling microscopy and confirmed to have atomic flatness [6]. As a result, EEM has been widely applied to the fabrication of ultraprecise mirrors used in synchrotron radiation facilities and EUVL [1].

However, further improvement of the figure correction system is needed because larger optical devices with more complicated figures are now required. For example, ultraprecise X-ray mirrors with a length of 400 mm have become necessary [7]. Ellipsoidal mirrors are also gaining increasing attention in the field of soft X-ray microscopy [8].

To improve the characteristics of stationary spot machining in EEM, we propose an improved method of flowing a fluid including particles. In particular, nozzle-type EEM utilizes a jet flow, which has been investigated in various fields such as water jet machining, water jet cleaning [9], and surface reforming with cavitation [10]. In these studies, the shape of the aperture and the structure of the channel in the nozzle are optimized to form a variable flow from the nozzle. The method used to simulate the fluid flow has also been improved. The behavior of a jet flow can be predicted and effectively used to develop functional nozzles.

In this study, we propose a nozzle structure to further improve the properties of stationary spot machining in EEM. The structure can concentrate the fluid after it flows from the nozzle aperture. A fluid simulation is carried out to clarify the advantageousness of the proposed structure. Then, the nozzle is fabricated and tested to confirm the simulation results.

## Methods

### Fluid simulations

In nozzle-type EEM, to transport particles to the workpiece surface and remove them from the surface, a high-shear flow is required on the surface. The removal area and removal rate depend on the velocity distribution of the fluid in contact with the surface. The shape of the distribution can be controlled by changing the nozzle specifications such as the width, velocity, angle, and stand-off distance, where the stand-off distance is defined as the length between the nozzle outlet and the workpiece surface.

**Figure 1:**
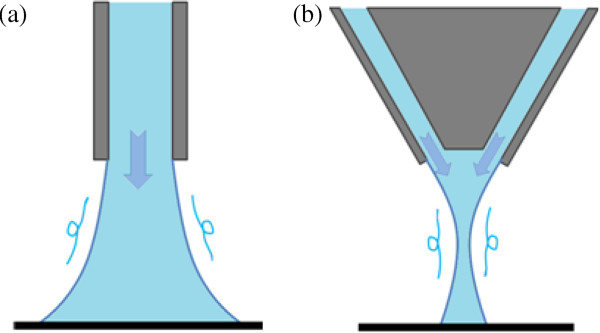
**Structure of nozzles used to generate high-shear flow on the workpiece surface in elastic emission machining. (a)** Straight-flow nozzle. **(b)** Focusing-flow nozzle.

In previous studies, the fluid channel of the nozzle was straight, and its aperture was rectangular or circular, as shown in Figure 1a [4]. The pressurized fluid flows from the nozzle toward the fluid in a tank. In this case, it is commonly considered that the flow diverges after exiting from the aperture since the jet flow is in a strongly turbulent state. To satisfy both the smallness and removal rate required in stationary spot machining, the stand-off distance is selected to be short. Minute stationary spot machining with a spot size of 500 μm in diameter has been realized for a stand-off distance of less than 300 μm [4].

In this study, the generation of a focusing flow is applied to EEM. Figure 1b shows the concept of a focusing flow. The channel structure inside the nozzle causes the flow to converge at a certain distance from the aperture. At the point of convergence, the maximum flow velocity is high, even far from the aperture. Furthermore, compared with the standard nozzle shown in Figure 1a, the velocity distribution on the workpiece surface is narrow, which enables a small stationary spot profile with a high removal rate in the case of long stand-off distances.

**Figure 2:**
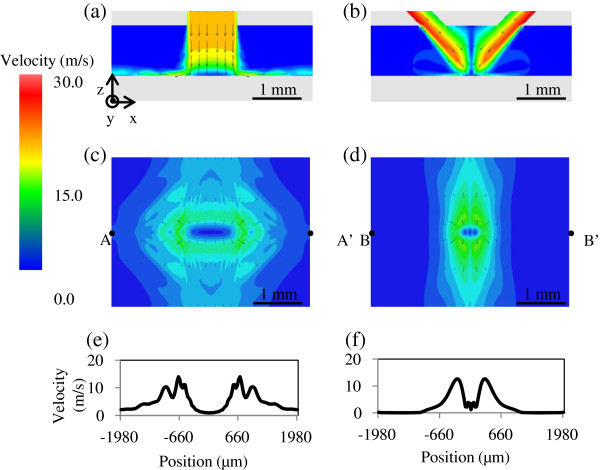
**Fluid simulation results showing the flow state of the jet.** Flow from the aperture to the workpiece surface in the case of a straight-flow nozzle and a focusing-flow nozzle. **(a)** Velocity distribution on XZ plane, straight-flow nozzle. **(b)** Velocity distribution on XZ plane, focusing-flow nozzle. **(c)** Velocity distribution on the plane, 1 μm from the workpiece surface, straight-flow nozzle. **(d)** Velocity distribution on the plane, 1 μm from the workpiece surface, focusing-flow nozzle. **(e)** Cross-sectional profile along A-A’ in **(c)**. **(f)** Cross-sectional profile along B-B’ in **(d)**.

To verify the effectiveness of the focusing flow, several fluid simulations were performed using a fluid simulation software (PHOENICS CHAM Co., London, England, UK)**.** The simulation parameters are listed in Table 1. In the case of a focusing-flow channel, the two streams meet after flowing from two apertures having a width of 500 μm and a thickness of 300 μm, as shown in Figure 1b. The angle between the two streams is 90°. In contrast, the straight-flow nozzle has a rectangular aperture with a dimension of 1 mm × 300 μm. The three-dimensional velocity and pressure distributions are calculated for both nozzles. The k-ϵ model included in the software is employed to calculate the turbulent flow [11]. To quantitatively analyze the effect of the channel structure, the flow speed at both nozzle apertures is set to be the same. Figure 2 shows the simulation results for the straight-flow channel and focusing-flow channel. The velocity distributions on the XZ plane including the center line are shown in Figure 2a,b. The velocity distributions on the plane, 1 μm from the workpiece surface, are compared in Figure 2c,d.

**Table 1:** Fluid simulation parameters

**Parameters**	**Model or values**
Turbulence model	k-ϵ model
Pressure	0.5 MPa
Atmosphere	Pure water at 20°C
Density	998.23 kg/m^3^
Viscosity	1.006 × 10^-3^ Pa s

As the flow approaches the workpiece surface, it undergoes significant changes in its velocity direction as it rotates from perpendicular to nearly parallel to the wall. This leads to a flow with a high-shear rate on the workpiece surface even when the stand-off distance is 1 mm. The fluid pressure is increased on the surface where the two flows meet at the center. Then, the direction of the main stream changes toward the *y*-axis.

From the viewpoint of machining, the velocity near the surface is an important evaluation factor. Figure 2e,f shows the cross-sectional profiles of the velocity distributions for the two types of nozzle. The profile is Gaussian-like with a full width at half maximum of 0.5 mm when the focusing-flow nozzle is used. In contrast, there are two peaks in the velocity distribution profile for the straight-flow nozzle. The distance between the two peaks is approximately 1 mm, which is the same as the nozzle aperture width. In EEM, the shape of the stationary spot profile depends on the distributions of the numbers of particles supplied to and removed from the workpiece surface. Since the diameter of the particles is as large as 2 μm in this study, the particles move along a streamline. A comparison of the two profiles indicates that a minute stationary spot profile can be obtained using the focusing-flow nozzle because the removal depth is basically proportional to the velocity close to the workpiece surface.

### Machining experiments

**Figure 3:**
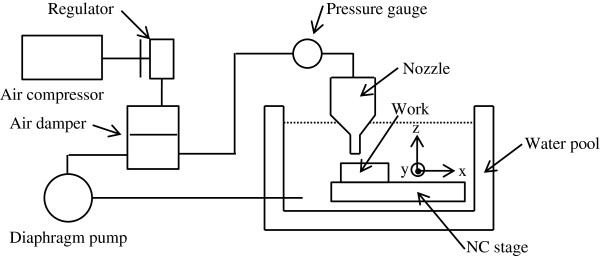
Schematic drawing of the nozzle-type EEM system used in this study.

Figure 3 shows a schematic drawing of the nozzle-type EEM system. In this system, the mixture fluid, which is composed of ultrapure water and fine powder particles, is supplied from the diaphragm pump to the nozzle head. The nozzle pressure is kept constant using the air compressor in the damper. The workpiece is set on the table in the tank. The table consists of an x-y stage, which controls the workpiece on the horizontal plane, and a z stage, which adjusts the gap between the nozzle and workpiece. The nozzle has a laminated structure consisting of two ceramic plates and a stainless steel sheet. The stainless steel sheet is cut according to the design of the channel structure.

We prepared and installed the two types of nozzle having the same channel structures as those used in the fluid simulations. Several stationary spots were machined on a quartz surface and measured using a microscopic interferometer with an area of view of 3.74 × 2.81 mm^2^ (ZYGO NewViewTM 700, Zygo Corporation, Middlefield, CT, USA)**.** The velocity was also adjusted in accordance with the simulation. The stand-off distance was varied from 0.4 to 1.8 mm. The experimental parameters are listed in Table 2.

**Table 2:** Experimental parameters in EEM process

**Parameters**	**Values**
Work material	Quartz glass
Powder particle	SiO_2_ 2 μm *φ*
Pressure	0.5 Mpa
Machining time	1 min
Solution concentration	3 vol.%
Stand-off distance	0.4, 0.6, 0.8, 1.0, 1.2, 1.4, 1.6, 1.8 mm

**Figure 4:**
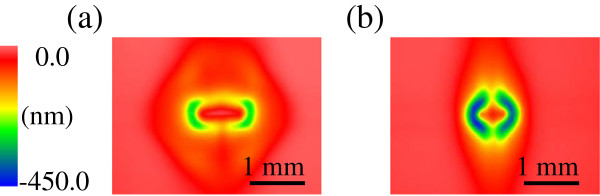
**Removal distributions of the stationary spot profiles obtained using the straight-flow and focusing-flow nozzles.** (**a**) Straight-flow nozzle. (**b**) Focusing-flow nozzle.

**Figure 5:**
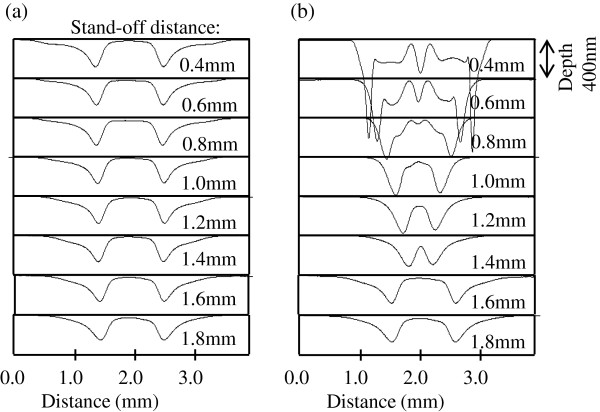
**Cross-sectional profiles of spots for stand-off distances from 0.4 to 1.8 mm. (a)** Straight-flow nozzle. **(b)** Focusing-flow nozzle.

**Figure 6:**
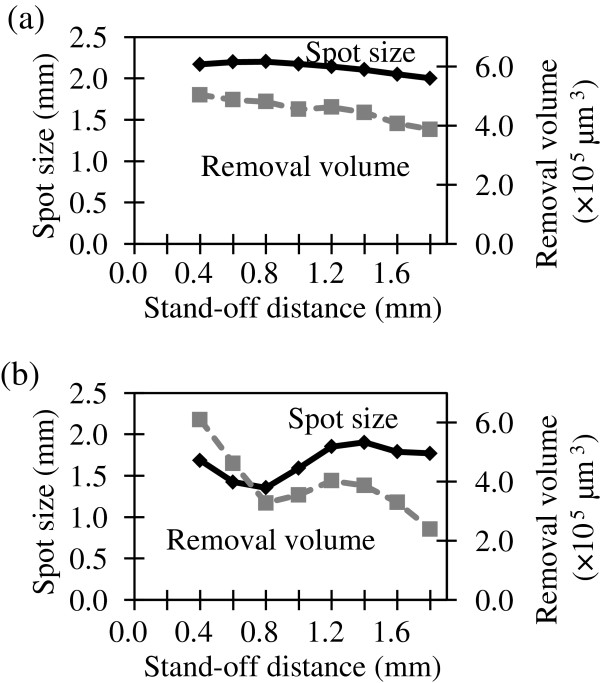
**Relationship between the stand-off distance, removal volume, and spot size. **Machining time is 1 min. **(a)** Straight-flow nozzle. **(b)** Focusing-flow nozzle.

Figure 4a,b shows the removal distributions of stationary spot profiles obtained using the straight-flow and focusing-flow nozzles, respectively, when the stand-off distance is 1 mm. Figure 5 shows the cross-sectional profiles of the spots for stand-off distances from 0.4 to 1.8 mm. The stand-off distance affects the shape, depth, and size of the spot. Figure 6 shows the relationship between the stand-off distance, removal volume, and spot size, where the diameter of the region including 80% of the total volume is defined as the spot size.

## Results and discussion

When the focusing-flow nozzle is employed, the spot size decreases with increasing stand-off distance from 0.4 to 0.8 mm. The minimum spot size is 1.3 mm at a stand-off distance of 0.8 mm, and as the stand-off distance increases, the spot size gradually increases. The results indicate that the spot size and removal rate can be controlled by simply adjusting the stand-off distance without changing the nozzle. On the other hand, when the straight-flow nozzle is used, the spot remains of the same size regardless of the stand-off distance. When a change in machining conditions is necessary, a nozzle with a different size must be installed [12].

**Figure 7:**
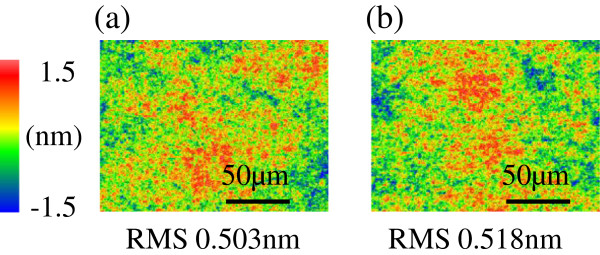
**Roughness of the surface before and after EEM processing using a focusing-flow nozzle. (a)** Before processing. **(b)** After processing.

Next, to evaluate the roughness of the EEM-processed surface, raster scanning was carried out on a quartz surface over a square area of side length of 5 mm before and after processing using the focusing-flow nozzle, as shown in Figure 7. The RMS values before and after processing are almost the same; thus, whereas the nozzle-type EEM is mainly employed for figure correction [4], the focusing-flow nozzle can also be used for the figure correction of advanced optical devices.

Finally, note that the stationary spot profiles in Figure 4a,b are in good agreement with the velocity distributions in Figure 2c,d, respectively. Thus, the shape of the stationary spot profiles can be predicted, which indicates that fluid simulators can be used for the further development of EEM nozzles suitable for figuring of various types of mirror.

## Conclusions

In this study, we proposed and experimentally tested the control of the shape of a stationary spot profile by realizing a focusing-flow state between the nozzle outlet and the workpiece surface in EEM. The simulation results indicate that the focusing-flow nozzle sharpens the distribution of the velocity on the workpiece surface. The results of the machining experiments verified those of the simulation. The obtained stationary spot conditions will be useful for surface processing with a spatial resolution higher than 1.3 mm.

In this study, the shape of the channel affected the machining parameters. The basic idea of controlling the shape of stationary spot profiles through not only the nozzle aperture size but also the channel structure can be widely applied to various EEM optical fabrication processes, particularly for advanced optics with a complicated shape.

## Competing interests

Both authors declare that they have no competing interests.

## Authors’ contributions

YT performed simulations and experiments. HM supervised the research work and helped amend the manuscript. Both authors read and approved the final manuscript.

## Authors’ information

YT is a graduate student, and HM is an associate professor at the University of Tokyo in Japan.
